# Role of Epigenetics in Modulating Phenotypic Plasticity against Abiotic Stresses in Plants

**DOI:** 10.1155/2022/1092894

**Published:** 2022-06-14

**Authors:** Fayaz Ahmad Dar, Naveed Ul Mushtaq, Seerat Saleem, Reiaz Ul Rehman, Tanvir Ul Hassan Dar, Khalid Rehman Hakeem

**Affiliations:** ^1^Department of Bioresources, Amar Singh College Campus, Cluster University, Srinagar, 190008 Jammu and Kashmir, India; ^2^Department of Bioresources, University of Kashmir, Srinagar, 190006 Jammu and Kashmir, India; ^3^School of Biosciences and Biotechnology, Baba Ghulam Shah Badshah University, Rajouri 185234, Jammu and Kashmir, India; ^4^Department of Biological Sciences, Faculty of Science, King Abdulaziz University, Jeddah, Saudi Arabia; ^5^Princess Dr Najla Bint Saud Al-Saud Center for Excellence Research in Biotechnology, King Abdulaziz University, Jeddah, Saudi Arabia; ^6^Department of Public Health, Daffodil International University, Dhaka, Bangladesh

## Abstract

Plants being sessile are always exposed to various environmental stresses, and to overcome these stresses, modifications at the epigenetic level can prove vital for their long-term survival. Epigenomics refers to the large-scale study of epigenetic marks on the genome, which include covalent modifications of histone tails (acetylation, methylation, phosphorylation, ubiquitination, and the small RNA machinery). Studies based on epigenetics have evolved over the years especially in understanding the mechanisms at transcriptional and posttranscriptional levels in plants against various environmental stimuli. Epigenomic changes in plants through induced methylation of specific genes that lead to changes in their expression can help to overcome various stress conditions. Recent studies suggested that epigenomics has a significant potential for crop improvement in plants. By the induction and modulation of various cellular processes like DNA methylation, histone modification, and biogenesis of noncoding RNAs, the plant genome can be activated which can help in achieving a quicker response against various plant stresses. Epigenetic modifications in plants allow them to adjust under varied environmental stresses by modulating their phenotypic plasticity and at the same time ensure the quality and yield of crops. The plasticity of the epigenome helps to adapt the plants during pre- and postdevelopmental processes. The variation in DNA methylation in different organisms exhibits variable phenotypic responses. The epigenetic changes also occur sequentially in the genome. Various studies indicated that environmentally stimulated epimutations produce variable responses especially in differentially methylated regions (DMR) that play a major role in the management of stress conditions in plants. Besides, it has been observed that environmental stresses cause specific changes in the epigenome that are closely associated with phenotypic modifications. However, the relationship between epigenetic modifications and phenotypic plasticity is still debatable. In this review, we will be discussing the role of various factors that allow epigenetic changes to modulate phenotypic plasticity against various abiotic stress in plants.

## 1. Introduction

**Figure 1:**
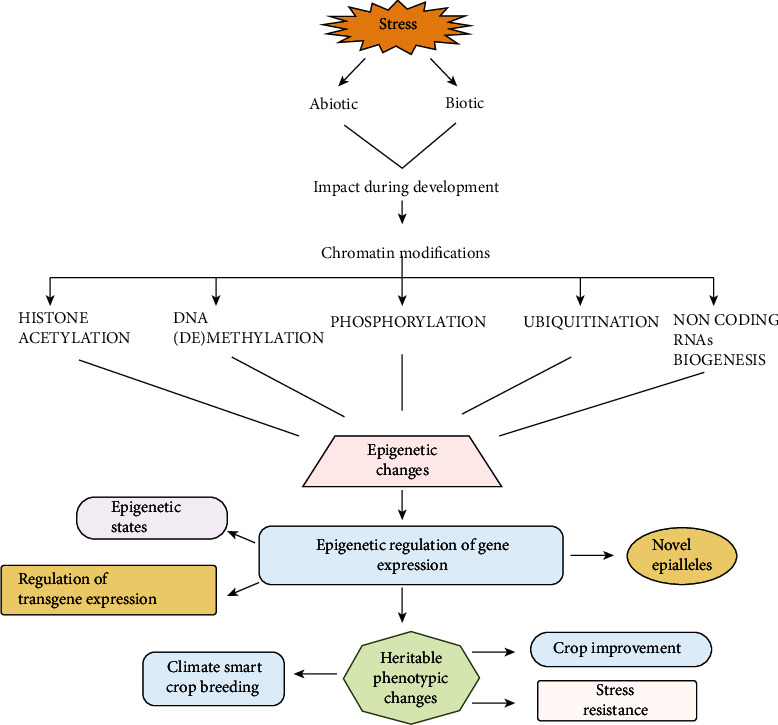
Epigenetic changes in plants under stress. Plants withstand various environmental stresses throughout their developmental stages and to overcome these challenges, epigenetic modifications play a vital role. These include covalent modifications of histone tails (acetylation, methylation, phosphorylation, ubiquitination, and the small RNA machinery). Epigenetic changes are the heritable phenotypic variations that are not always due to specific DNA sequence alterations, but the epigenetic regulation that involves certain chemical modifications at the molecular level which can alter the gene expression. DNA and chromatin modifications at the epigenomic level affect gene expression and play a prominent role in unveiling phenotypic responses against external stimuli. Epigenetic changes are reversible and heritable to control gene expression without any change in the DNA sequence. Epigenetics holds immense potential in crop improvement strategies, climate-smart breeding and stress resistance by choosing the favorable epigenetic states, formulation of novel epialleles, and regulation of transgene expression.

The immobile lifestyle of plants exposes them to different types of biotic and abiotic stresses. Drought, salt, severe temperatures, nutritional deficits, heavy metal toxicity, and UV radiation are some of the most common abiotic stressors. Agricultural production is being threatened by these unfavorable conditions. To adjust under such variable conditions, plants undergo consistent changes at the physiological and molecular levels. Epigenetic changes, which increase plant longevity by improving their tolerance to stress, provide these productive and viable controls. Various studies have concluded that heritable phenotypic variations are not always due to specific DNA sequence alterations, but the epigenetic regulation that involves certain chemical modifications at the molecular level can alter the gene expression [1, 2]. Among the variations induced by normal DNA sequence variations, epigenetic changes are the changes resulting from hereditary changes in gene function acquired during mitotic/meiotic cell division. Nowadays, epigenetics is defined as the changes in the genome that occur due to heritable chemical modifications instead of usual changes in the DNA sequence [3]. Several epigenetic changes are observed, including modifications to DNA bases, histones, and noncoding RNAs. In addition, transcription, replication, chromosome condensation/segregation, and DNA repair mechanism are all affected by posttranslational modifications. DNA and chromatin modifications at the epigenomic level affect gene expression and play a prominent role in unveiling phenotypic responses against external stimuli [4] (Figure 1). This is all due to chromatin, a highly organized and compact complex of DNA and associated proteins. Nucleosome consists of around 200 base pairs of DNA wrapped around octamers of core histone proteins H2A, H2B, H3, and H4 [5, 6].

Epigenetics refers to the study of heritable phenotypic modifications that do not involve alterations in DNA sequence [7]. Plant phenotypic diversity under stress is often linked to DNA sequence variations. Recently, it was shown that epigenetic alterations can contribute to phenotypes alone or in combination through modulating gene expression in response to stress. When natural populations are subjected to changes in environmental circumstances, their performance might differ. The mechanisms behind one plant's stress response may differ from those underlying another [8].

Environmental factors continuously shape postembryonic plant development, resulting in a high level of phenotypic plasticity. Although plants cannot escape their surroundings, they adapt to the changing and unfavorable growth conditions. The control of gene expression patterns and epigenetic regulation work together to promote metastable changes in gene activity. All these factors help the plants to cope to the unpredictable environments [9]. A potential link between embryonic environmental factors and diseases is inherent in epigenetics which suggests that gene expression is controlled by reversible, heritable changes rather than inevitable changes to DNA sequences. Most epigenetic mechanisms regulate gene expression through DNA methylation, histone modifications, or small noncoding RNAs. Plants are an ideal system to study epigenetic processes. Plant reproductive development is associated with DNA methylation changes. Most of the studies on DNA methylation come from *Arabidopsis thaliana*; however, all the plant genomes undergo methylation where some pathways are known to predominate while some are defective. The family of DNA methyltransferases (DNMTs) catalyzes a process that results in a methyl group being attached to the cytosine of DNA [10]. The covalent addition of acetyl groups to lysine residues modifies the histones with increased transcription [5]. Endogenous hormonal signaling also plays a role in histone modification patterns. Positively charged lysine residues when acetylated undergo neutralization of charges. The interactions between the histone and DNA are weakened, and the opened chromatin is more accessible to regulators [11]. Histone acetyltransferases (HATs) catalyze lysine or serine acetylation, and histone deacetylases (HDACs) are responsible for reversing this process. Histone acetylation is usually linked with gene expression, while deacetylation is linked with gene repression [12]. H3 phosphorylation regulates gene expression and participates in chromosome condensation/segregation [13]. Both plants and metazoa phosphorylate conserved residues on histone H3, such as Thr3, Ser10, Thr11, and Ser28 during interphase or mitosis, resulting in different mechanisms for activating transcription [14, 15]. Crop improvement strategies can be designed using epigenetics, such as selecting the most favorable epigenetic states, generating novel epialleles, and regulating the expression of transgenes. Epigenetic factors are considered indicators of the transgenerational plasticity in plants [10]. Epigenetics also acts as a stress memory in plants, allowing them to cope more effectively with future stress [1]. An example of epigenetic regulation in plant stress response is the phenomenon of vernalization where plants have a memory of prolonged exposure to low temperature in the winter to flower in the spring [2].

## 2. Types of Epigenetic Changes in Plants

Plants and animals both rely on epigenetic mechanisms during their life cycles. Chromatin comfirmation is vital for the proper regulation of genes and genome activity. DNA methylation and histone modification in plants are associated with the modulation of stress-responsive genes. Abiotic stress can cause chromatin regulators such as acetylation, methylation, and phosphorylation to regulate gene networks that respond to stress [16, 17]. It has been reported that histone modifications such as acetylation, phosphorylation, and ubiquitination enhance gene transcription, while biotinylation and sumoylation suppress gene expression [4].

**Figure 2:**
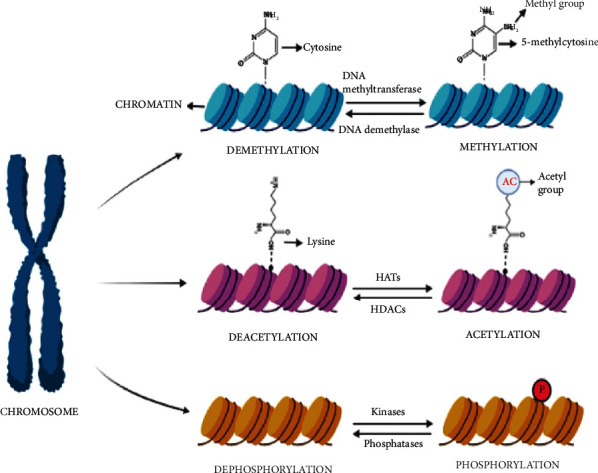
Various epigenetic modifications include acetylation, (de)methylation, and phosphorylation. DNA methylation involves the addition of a methyl (-CH_3_) group to the fifth position of cytosine known as methylcytosine (5-mC). This process is carried by DNA methyltransferases while the demethylation process is aided by DNA demethylase. Acetylation, i.e., the addition of negatively charged acetyl group to lysine residues on histone proteins is regulated by two opposing enzymes, i.e., histone acetyltransferases (HATs) and histone deacetylases (HDACs). Acetyl group addition is catalyzed by HATs while the removal of acetyl groups is catalyzed by HDACs. Phosphorylation is one of the important histone modifications. Histone phosphorylation plays a role in DNA repair, synchronization of chromosome segregation, and cell division. Histone tails can be phosphorylated by various protein kinases and dephosphorylated by phosphatases. All the histone modifications lead to the regulation of gene expression.

Plants undergo epigenetic-based programming during growth, development, and under stress conditions, which results in the regulation of gene expression without modification of DNA sequences [18]. Acetylation, methylation, phosphorylation, ubiquitination, and sumoylation are the various posttranslational modifications of histones. It has been reported that various environmental stimuli trigger dynamic epigenetic modifications, which is an essential mechanism for signal-induced transcription [11] (Figure 2).

## 3. Acetylation

Histone acetylation occurs when lysine residues on the histone proteins are added with a negatively charged acetyl group. A process initiating histone acetyltransferases (HATs) and histone deacetylases (HDACs) is regulated by two opposing enzymes. HATs catalyze acetyl group addition, while HDACs catalyze its removal. A decrease in histone acetylation results in a chromatin structure that allows transcription to happen more freely because of its reduced electrostatic affinity with DNA [19]. Various chromatin proteins and transcription factors interact with HAT or HDAC proteins to control gene expression at specific genomic or chromatin regions as a consequence of changes in cellular signaling [20].

Acetylation of histones is a reversible and dynamic process. It was proposed more than 40 years ago that transcriptional activity is correlated with histone acetylation. The highly basic nature of amino tails is attributed to the high content of lysine and arginine amino acids. Conserved lysine residues are acetylated which neutralizes the positive charge of histone tails which results in reduced affinity for negatively charged DNA that in turn promotes the accessibility of chromatin to transcriptional factors [21]. Lysine acetylation plays an important role in epigenetic processes. It has evolved as a key posttranslational modification that can be found at multiple places throughout the cell [22]. Lysine acetylation is one of the major protein posttranslational modifications (PTMs) that is important for many enzymes catalyzing intracellular metabolism which implies that protein acetylation has an important role to play in cellular functions [23]. HATs in plants are divided into four classes: cAMP-responsive element-binding protein (CBP), general control nondepressible 5- (GCN5-) related acetyl transferase (GNAT), MOZ-YBF2/SAS3-SAS2/TIP60 (MYST), and TATA-binding protein associated factor 1 (TAF1). A variety of HATs are involved in acetylating specific lysine residues, for instance, HAG1 and HAG2 HATs from the GNAT class catalyze H3K14 and H4K12 acetylation, respectively. HAM1 and HAM2 belong to MYST class HATs acetylate H4K5. As distinct HAT molecules recognize acetylated lysines of histone with different reader proteins, their specific roles in gene regulation can be reflected by enzymatic specificities [24]. Based on the subcellular distribution, HATs are grouped into two categories. In the cytoplasm, type B HATs catalyze the acetylation of histone H4 at lysine 5 and 12, which occurs before the incorporation of the histone into newly replicated chromatin. HAT of type B has been described in maize and functions as a heterodimer. In the nucleus, type A HATs play a role in regulating chromatin assembly and gene transcription by acetylating nuclear histones [21]. The HDACs are divided into three different families. The first family is identical to yeast: reduced potassium deficiency 3 (RPD3), the most widely studied and found throughout eukaryotes. The second family, HD-tuins (HDT), is only present in plants and is originally discovered in maize. The third family, sirtuins, is structurally distinct and is homologous to the yeast silent information regulator 2 (Sir2), which is a nicotinamide adenine dinucleotide- (NAD-) dependent enzyme [25].

Both HDACs and HATs interact with protein complexes as corepressors and coactivators of transcription or to modulate DNA accessibility to various types of machinery through associations with chromatin remodelers. Detailed studies have been conducted on the HAT and HDAC superfamilies in *Arabidopsis*, rice, and tomato. The HATs and HDACs play essential roles in plants' response to abiotic and biotic stresses. For example, they specifically increase acetylation of histone H4K5 and H3K9 in the promoter and transcribed region of the maize C (4)-Pepc gene in response to light. In addition to regulating temperature, histone acetylation is instrumental in the development of plants [26]. The levels of acetyl-CoA and NAD^+^ in the cells play an important role in the acetylation and deacetylation processes and are linked to the activity of HATs and HDACs [27]. Plants with different levels of HAT gene expression display different drought-resistant traits. HAT genes *TaHAG2*, *TaHAG3*, and *TaHAC2* were induced under drought stress in a wheat variety called BN207, but not in other varieties with lower drought resistance. Li et al. [28] reported that, in soybean, drought treatment decreased expression levels of nine *GmHDAC* genes (*GmHDA6*, *GmHDA8*, *GmHDA13*, *GmHDA14*, *GmHDA16*, *GmSRT2*, *GmSRT4*, *GmHDT2*, and *GmHDT4*).

## 4. DNA Methylation

DNA methylation is the covalent addition of a methyl (-CH_3_) group to the fifth position of cytosine known as methylcytosine (5-mC) ring in presence of enzymes DNA methyltransferases. It is a heritable and reversible process based on genetic and cellular modification mechanisms like transposon silencing, tissue-specific gene expression, and genome balance after polyploidization. DNA methylation corresponds with transcriptional silencing and typically takes place in DNA sequences containing cytosines adjacent to a guanine base (called a CpG site) [29]. In addition to regulating gene expression, growth, development, and protection against environmental stresses, DNA methylation is important in stabilizing genomes. Cytosine methylation in plants can occur in all contexts of cytosine (CG, CHG, and CHH, where H = A, C, or T). Different methyltransferases (that function by utilizing S-adenosyl-l-methionine as a methyl donor) are responsible for DNA methylation, while enzyme-mediated base excision repair (BER) is responsible for active DNA demethylation. Different processes initiate, maintain, and remove cytosine methylation in distinct genomic regions of the plant genome. The RNA-directed DNA methylation (RdDM) pathway is involved in de novo cytosine methylation. This pathway utilizes small-interfering RNAs (siRNAs), scaffold RNAs, and many accessory proteins. The maintenance of cytosine methylation is dependent on various DNA methyltransferases. A set of enzymes (bifunctional 5-methylcytosine DNA glycosylases–apurinic/apyrimidinic lyase (APE1L)) begin the demethylation process *via* the BER route during inactive DNA demethylation [30]. In plants, DNA methylation is crucial for development and to counter biotic and abiotic stresses [31]. Reportedly, plants infected by pathogens show genome-wide DNA methylation changes. Roots of soybean and *Arabidopsis thaliana* infected by cyst nematodes showed widespread DNA hypomethylation [32, 33]. Recent research has suggested the importance of DNA methylation in mediating plant stress responses to abiotic environmental stimuli [34]. During inorganic phosphate starvation, rice plants generate more than 100 differentially methylated regions (DMRs) spanning mostly CHH hypermethylated transposons near genes responding to stress and referred to as Pi-starvation-induced (PSI) genes. Salinity stress in *A. thaliana* changed the DNA methylation process, which could be passed on to the next generation primarily *via* female germlines [35]. Apart from its role in stress resistance and development, DNA methylation suppresses harmful DNA sequences, such as retroviral genes, which have been incorporated into host genomes during evolution [10].

DNA methylation contributes significantly towards modifying the genome of plants and thus increases their adaptability and yield as these changes get inherited to the next generation of plants. Studies have shown that there is variable DNA methylation within and among the plant in their natural environments. DNA methylation has been found to influence several plant traits like flowering time, seed dormancy, and yield of agronomically important plants, and therefore, epigenetic changes can help in domestication and evolutionary processes [36]. Modulation during DNA methylation in plants can be of vital significance to breeders and molecular biologists to decide the induction of selective variations in plants through tissue culture and transgenic approaches. An increased (hypermethylation) and decrease (demethylation) level of DNA methylation is seen in response to stress. In comparison to the plants grown in normal soil conditions, rice and mangrove plants showed hypermethylation when grown under high salinity. On the other hand, tobacco infected with tobacco mosaic virus (TMV) showed hypomethylation, which requires specific expression of 31 stress-related genes. Drought conditions led to CG hypermethylation in the pea genome [37].

## 5. Phosphorylation

In addition to methylation and acetylation, phosphorylation is one of the important histone PTMs. Histone phosphorylation plays a role in DNA repair (ɣH2AX) and synchronization of chromosome segregation and cell division [9]. The phosphorylation of histone H2A(X) during DNA damage cellular response is responsible for the delimitation of large chromatin domains surrounding the DNA damage site. Various protein kinases and phosphatases can phosphorylate and dephosphorylate the acceptor site present at four nucleosomal histone tails, respectively. Several residues in histones can be phosphorylated, including serine, threonine, and tyrosine. Many phosphorylated histone residues are important for gene expression, e.g., phosphorylating serines 10 and 28 of H3 and serine 32 of H2B appears to be linked to transcription of genes responding to epidermal growth factor (EGF) [38]. Phosphorylation is also correlated to chromosome condensation. When the cell enters mitosis, the histone H3 is highly phosphorylated. This phosphorylation is considered to be a crucial step in chromatin condensation and compaction which is an essential criterion for chromosome congression and segregation through mitosis and meiosis [39]. Increased salt tolerance in tobacco and *Arabidopsis* is attributed to phosphorylation of histone H3, S10, and acetylation of histone H4 [40]. In *Arabidopsis*, MAP kinase MPK3, which is an important component of stress defense signaling, phosphorylates histone deacetylase HD2B, leading to reprogramming of defense gene expression and innate immunity with the result of intranuclear compartmentalization of HD2B [41]. It has been reported that *abiotic stress response* in *Arabidopsis led to phosphorylation of decapping factors.* DCP1 is phosphorylated by MAP kinase 6 (MPK6), and this phosphorylated DCP1 promotes dimerization and association with DCP2 and DCP5. When drought stress is present, this interaction enhances decapping activity [42]. In *A. thaliana*, osmotic stress increases the phosphorylation of histone H3 threonine 3 (H3T3ph) in pericentromeric regions where it is believed to help maintain heterochromatin structure. There is also evidence that H3T3ph acts as a repressor on gene expression by antagonizing H3K4me3 during osmotic stress [43].

## 6. Ubiquitination

Ubiquitination is the process in which a ubiquitin molecule covalently attaches itself to a target molecule. All eukaryotes contain ubiquitin, a 76-amino-acid residue that is highly conserved among plants, with only two and three differences between yeast and human homologs. Signaling molecules like ubiquitin regulate cellular homeostasis and trigger a variety of cellular processes. The monoubiquitination process involves linking the target molecule with the C-terminal residues of one of the eight ubiquitin residues (Lys6, Lys11, Lys27, Lys29, Lys33, Lys48, Lys63, and Met1). On the other hand, the attachment of multiple ubiquitin molecules to other lysine residues of the same substrate is called multiubiquitination [44]. Ubiquitin-conjugating enzyme E1 transfers active ubiquitin to ubiquitin-conjugating enzyme E2, and then E3 (ubiquitin ligase) deposits the active ubiquitin onto the target protein (usually on a lysine residue). Polyubiquitin substrates are degraded *via* 26S proteasome while monoubiquitination (ub) or short ub-chains are usually degraded *via* the lysosome. A group of proteins known as ubiquitin-deconjugating enzymes are responsible for deubiquitination. The deubiquitinase superfamily (DUB), one of the biggest superfamilies works antagonistically to the action of E3 ligases. The processes of ubiquitination and deubiquitination play a vital role in many processes such as cell homeostasis, signal transduction, transcriptional gene regulation, protein degradation, and endocytosis [45]. Ubiquitination can regulate transcription by being either an active or repressive marker. Genes with trimethylated H3K4 and H3K36 (H3K4me3 and H3K36me3) and those with monoubiquitinated H2B (H2Bub) are often active. A RING E3-ligase called HUB1 and HUB2 is responsible for H2B histone monoubiquitination. In addition to controlling flowering time, the cell cycle, seed dormancy, and the circadian clock, these ligases also protect against necrotrophic fungal pathogens [46]. Epigenetic control is also extended by ubiquitination and deubiquitination of histone proteins. A plant cell's chromatin is turned on by monoubiquitination for downstream cellular activation processes. Upon deubiquitination by deubiquitinating proteins, ubiquitin is detached from histones and gene expression is repressed and downstream processes are inactivated [18]. In *Arabidopsis*, the transcription factor, WRKY34, promotes the expression of the ubiquitin E3 ligase, *CULLIN 3A*, thereby resulting in proteasomal degradation of FRIGIDA (FRI) and thereby leading to the accumulation of long noncoding RNA (lncRNA) and *cold-induced long antisense intragenic rna* (*ColdAIR*) in the late phases of vernalization. High *ColdAIR* levels reduce H3K4me3 in *FLC (flowering locus C)* facilitating flowering after vernalization [47]. U-box E3 ubiquitin ligase TaPUB1 enhances salt stress tolerance in wheat by upregulating the expression of genes related to ion channels and genes that improved the antioxidant capacity of plants under salt stress [48]. Moreover, the membrane-bound E3 ubiquitin ligase gene BnTR1 isolated from *Brassica napus* contributes to the development of thermal resistance in plants by regulating calcium channels and heat shock proteins [49]. Ubiquitin-specific protease (USP) protein families are involved in protein deubiquitination. In *Arabidopsis,* these participate in ABA signaling, drought and salt tolerance, nutrient deficiency response, and immunity regulation [50].

## 7. Small RNA Machinery

The plant genome encodes an array of small RNAs that are involved in the development, reproduction, and reprogramming of the genome, besides contributing to its phenotypic plasticity. Small RNAs play a significant role in both defense and epigenetic responses, according to recent research. DICER-like proteins (DCLs) help create small RNA molecules by synthesizing 21–24 nucleotide RNA molecules. In plants, small RNAs are divided into microRNA (miRNA) and small interfering RNA (siRNA) by their origin, structure, and pathways they regulate. Endogenous siRNAs in plants can be classified into several major groups, i.e., hairpin-derived siRNAs (hp-siRNAs), transacting siRNA, natural antisense siRNAs (natsiRNAs), secondary siRNAs, and heterochromatic siRNAs (hetsiRNAs). Plants modify all small RNAs at the 3′ end by 2′ omethylation, including microRNAs. To enhance stability and prevent degradation following 3′ uridylation, modification is essential. A miRNA participates in posttranscriptional gene silencing in plants by cleaving transcripts or repressing translation. Many siRNAs are involved in PTGS, but a majority of them are involved with RNA-directed DNA methylation (RdDM) and transcriptional gene silencing (TGS) [37, 51, 52]. RdDM is the de novo methylation caused by double-stranded RNA (ds-RNA) molecules. The interrelation between RdDM and RNA interference (RNAi) suggests that small RNAs guide cytosine methylation. RdDM pathways help in adaptation responses to various stresses, maintaining genome stability and regulation of development [37]. Small RNAs and long noncoding RNAs (lncRNAs) have come out as key regulators of chromatin structure in eukaryotic cells. In addition to RNA degradation, translational suppression, chromatin modification, and RNA interference (RNAi) pathways, small RNAs are also involved in targeted gene expression. Nuclear RNAi pathways repress transcription through histone or DNA methylation. Using *A. thaliana* as a model system, scientists first demonstrated that DNA methylation of target genes, as well as posttranscriptional gene silencing, was associated with small interfering RNA (siRNA) production, linking RNA-directed DNA methylation to the RNAi pathway [53]. Researchers have reported that a 24-nt siRNA in Arabidopsis is involved in downregulating P5C dehydrogenase (P5CDH) expression via mRNA cleavage, resulting in a decrease in proline degradation and an increase in prolines accumulation and salt stress tolerance [4].

## 8. Epigenetic Modifications under Different Stress Conditions

The persistent transmission of many DNA sequence alleles has long been linked to the heritable foundation of complex characteristics acquired or maintained in plants under stress [54]. Allelic DNA variation may be altered by mutations, influencing the genetic architecture and how these alleles are expressed. The recent discovery that diversity in chromatin states is abundant in experimental and wild populations and may constitute an additional source of phenotypic variation has called into question the traditional idea that DNA alleles are the only cause of phenotypic variation [55, 56]. A plant's chromatin state can be altered rapidly and irreversibly by DNA methyltransferases inserting methyl groups in cytosine, acetylation and methylation of N-terminal histone tails to promote chromatin remodeling, and small RNA mechanisms that prevent population diversification due to excessive rearrangements [57, 58]. Epigenetic alterations are those that modify DNA activity without changing its underlying nucleotide structure [59]. Because epigenetic alterations may be triggered by environmental cues and passed down to future generations, they may add another layer of complexity to heritable phenotypic diversity and the evolutionary potential of wild populations [60]. Various epigenetic processes and the associated genes under different stress conditions are mentioned in Table 1. Epigenetics plays a vital role in crop improvement. The epigenetic modifications induced in various crop species have resulted in crop improvement.  Table 2 summerises some of these modifications.

## 9. Epigenetic Modifications under Salt-Induced Stress

Environmental pressures cause DNA methylation to be either hyper or hypo. Research studies suggest that epigenetic processes play a major role in altering genes under adverse conditions. Under salt-induced stress, methylation of promoters and gene bodies helps control gene expression in a genotypically and organ-specific manner. It has been demonstrated that salt stress manifests itself in soybean by altering the expression of several transcription factors. In their study, Song et al. [61] speculated that DNA methylation and histone modifications could function collectively to affect stress-inducible genes. Furthermore, Ferreira et al. [62] suggest that salt stress may alter the expression of DNA demethylases leading to hypomethylation. Likewise, the salt-resistant wheat cultivar SR3 as well as its progenitor HBP1 showed opposing changes in cytosine methylation patterns when exposed to salinity-induced stress. A specific gene for demethylation was found to be effective during epigenetic processes in rice roots in response to salinity stress [63]. Salt stress may cause changes in the expression levels of high-affinity potassium transporters whose expression is controlled by genetic and epigenetic processes in wheat [64].

Changes in chromatin structure play a key role to impart salinity tolerance in many crop plants. According to Kaldis et al. [65], the transcriptional adaptor ADA2b is responsible to induce hypersensitivity in *Arabidopsis thaliana* under salt stress. Consequently, salt stress impacts both genome-wide DNA methylation and histone changes, and both these processes are related to one another for enabling coordinated salt stress response [66]. DNA methylation was shown to be higher in the shoots of salt-sensitive and salt-tolerant wheat genotypes, ranging from 30 to 40%, as compared to that in roots [67, 68]. Under salt stress, DNA methylation was shown to be higher in sensitive genotypes when compared to intolerant genotypes in rape seeds [69].

Under abiotic stressors such as salt, DNA demethylation in tobacco generates the NtGPDL gene (glycerophosphodiesterase-like protein). Likewise, salt stress in rapeseed induces cytosine methylation at CpCpGpG sites increased, with methylation and demethylation. The enzymes involved in the process are histone deacetylases (HDACs) and histone acetyltransferases (HATs) [57]. Plants exhibit altered responses to salt when HDAC activity occurs, and HDAC toxicity inhibits the activity of HAT complexes [20, 70]. There was a discovery that sRNAs were associated with hypermethylated areas [71].

## 10. Epigenetic Changes under Drought-Induced Stress

Research on the stress biology of plants has mostly focused on elucidating regulatory mechanisms at the complex molecular levels in plants exposed to a variety of environmental constraints. Drought-induced gene expression is primarily regulated by histone alterations and nucleosome density in the promoters and ORFs of the OR genes [72]. Histone changes such as H3K4me3 and H3K9ac were found in the Rd20 and Rd29A regions under drought stress compared to optimal conditions [73].

A thorough examination of the postdrought stress healing process demonstrated rapid deacetylation at the H3K9ac sites, preceded by the ultimate clearance of RNA polymerase II from such areas. Rd29A, Rd20, and *Arabidopsis* galactinol synthase were the genes implicated in such substantial chromatin shifting (AtGOLS2). However, H3K4me3 was eliminated slowly than H3K9ac with larger rehydration dosages [1]. An elevation in H3K4 trimethylation and H3K9 acetylation on the gene promoter, as well as H3K23 and H3K27 acetylation on the coding sections, was shown to be crucial for drought-induced transcription of stress-sensitive genes in *A. thaliana* [74]. By boosting H3K4me3 modification, the *Arabidopsis* trithorax-like 1 HMT increased the overexpression of the gene encoding the ABA biosynthesis enzyme, 9-cis-epoxy carotenoid dioxygenase, notably under drought stress [75].

DNA methylation demonstrates tissue selectivity during drought stress. Drought caused a total of 12.1% methylation changes in *Oryza sativa*, which were accounting across different tissues, genotypes, and life stages. The total DNA methylation rate in roots was lower than those in leaves at the same developmental period, indicating that roots play a key role in water deficiency [76]. The connection between DNA methylation and drought stress resistance has been demonstrated in rice cultivars IR20, a drought vulnerable variety, exhibits hypomethylation under drought conditions, while the resistant variants exhibit hypermethylation [77].

## 11. Epigenetic Modifications under Heat-Induced Stress

Heat stress is principal to abiotic stress in plants, with distinct negative effects on plant development, physiology, and metabolism [78]. Heat, like other stressors, causes epigenetic changes in plants. Such adaptations enable the plants to cope up with heat stress. Several studies have suggested the role of these changes in plants against heat stress. Heat stress causes greater methylation and frequent occurrence of homologous recombination in *Arabidopsis* plants [79]. In response to heat stress, DRM2, nuclear RNA polymerase D1 and NRPE1 overexpression may result in enhanced genome methylation in *Arabidopsis* [80]. Cork oak (*Quercus suber* L.) cultivated at 55°C shows an increase in global methylation [81]. In *Brassica napus*, the heat-sensitive genotype exhibits higher DNA methylation levels than the heat-tolerant genotype following heat stress [82].

In developing rice seeds, mild heat stress at 34°C for 48 hours reduces the DNA methylation level of fertilization-independent endosperm1 (OsFIE1), a member of polycomb repressive complex 2 (PRC2), and represses the transcript abundance of OsCMT3, which may contribute to OsFIE1 misregulation [83].

The expression of lysine-specific histone demethylase 1 (LSD1), which is involved in the demethylation of histone H3 lysine 4 (H3K4) me1/2, ribosomal RNA FtsJ-like methyltransferase, has increased in heat stress primed second-generation plants. This demonstrates that some epigenetic markers stimulate genes in the offspring of primed plants to produce tolerance. [84].

A histone variant H2A.Z induces transcriptional alterations in stress-responsive genes at high temperatures [16]. In *Arabidopsis thaliana*, mutations in the GCN5 gene, which encodes histone acetyltransferase, reduced transcriptional activation of heat stress-sensitive genes including HSAF3 and MBF1c resulting in Arabidopsis thaliana thermal vulnerability [85]. Furthermore, brief heat stress stimulated heat shock protein 101 (HSP101) expression in *Arabidopsis*, but expression dropped after repeated brief stress treatments, indicating that the plant epigenome is suited to recover from temperature stress [86].

## 12. Epigenetic Modifications under Cold-Induced Stress

Cold stress has been recognized as a major environmental issue limiting agricultural growth and productivity especially in steep terrain [87]. Reduced temperature impairs plant development physiology by causing chilling ailments such as photosynthetic apparatus damage, chlorosis, tissue death, loss of membrane integrity, and eventually wilting [88]. Under cold stress, the expression of epigenetic regulators fluctuates [1]. During cold acclimation, *Zea mays* showed increased expression of histone deacetylases (HDACs). As a result, the lysine residues on the histone subunits H3 and H4 were deacetylated [89]. HOS15, a WD40-repeat protein, functions to control gene expression through histone deacetylation in chromatin. HOS15 interacts specifically with and promotes deacetylation of histone H4, indicating that chromatin remodeling plays an important role in gene regulation in plant responses and tolerance to abiotic stresses [90]. HOS15 interacts with histone deacetylase 2C (HD2C) and both proteins together associate with the promoters of cold-responsive COR genes, COR15A and COR47. Cold-induced HD2C degradation is mediated by the CULLIN4- (CUL4-) based E3 ubiquitin ligase complex in which HOS15 acts as a substrate receptor [91].

An alternative splicing pathway involving histone demethylase, Jumonji C domain-containing gene (JMJC5) was discovered in *Medicago truncatula* [92]. When *Arabidopsis* plants were exposed to low-temperature stress conditions, they exhibited enhanced H3T3ph and H3K4me3 at genome level but low histone H3 occupancy at the pericentromeric regions [93]. Throughout the chilling and freezing phases, rapid changes in cytosine methylation occurred [94]. miR6445a stability was modulated by signature methylation in *P. simonii* subjected to cold, salt, osmotic, and heat stressors [95]. As a result of cold treatment, *Gossypium hirsutum* exhibited a decrease in DNA methylation and an increase in the expression of trehalose-6-phosphate synthase-like gene, associated with plant defense [96]. The DNA methylation of cold-response defense genes, including HbICE1, was altered in *Hevea brasiliensis* following cold treatment [97], suggesting that low-temperature response machinery involves regulation of defense-related genes via DNA methylation changes. Under brief cold stress, patterns of whole-genome demethylation were also discovered in Zea mays, allowing transposons and stress-responsive genes to be modulated [98].

## 13. Conclusions

A rapidly growing population and climate change have presented several challenges to agricultural and food production worldwide. We might be able to achieve sustainable agricultural output if we understand plant stress responses and create new tactics of protecting plants. Through epigenetic adjustments, plants may be able to adapt under variable biotic and abiotic stress factors. Cellular alterations, for instance, DNA modifications, chromatin changes, small RNA pathways, and DNA methylation, all work together to regulate the expression of stress-responsive genes in plants under different stress conditions. Various abiotic stresses can now be controlled epigenetically to enable diverse plant species to adjust under different sets of conditions. Epigenetic methods have been applied in a variety of crops. The use of epigenetics may be another method of developing defensive mechanisms in plant species exposed to a variety of environmental challenges. There is certainly a need for more research in this promising field to make it viable and widely applicable. The development of markers to track epigenetic changes, the durability of priming, and advances to understand multiple stresses that plants encounter in their lifetime are all essential to optimize its application in crop protection programs in the future.

## Figures and Tables

**Table 1:** Different epigenetic processes and the associated genes.

Epigenetic process	Gene/enzyme	Function	Plant	Reference
DNA methylation	Asr1 and Asr2	Drought stress tolerance	Tomato	[99]
Demethylation and hypomethylation	Glyma11g02400	Salinity tolerance	Soybean	[61]
Histone modification	OsHAM701	Drought stress tolerance	Rice	[28, 100]
Histone modification	OsHAC701	Heat stress tolerance	Rice	[101, 102]
Histone modification	AtABO1	Drought and oxidative stress tolerance	Arabidopsis	[103, 104]
Histone modification	AtATX1/HvTX1	Drought stress tolerance	Arabidopsis and barley	[75, 105]
Hypomethylation	NtGPDL	Cold tolerance	Tobacco	[106, 107]
MicroRNA	miR170, miR171 and miR172	Drought tolerance	Wheat/millet	[108, 109]

**Table 2:** Epigenetics for crop improvement.

Species	Epigenetic modification	Crop improvement	Reference
Arabidopsis and tomato	MicroRNA	Enhanced plant vigor and phenotypes	[102]
Arabidopsis, rice and maize	DNA methylation	Development of new molecular markers	[81]
Arabidopsis	Induced expression of miR156 and miR396	Salt stress response	[110]
Arabidopsis	Induced expression of miR393, miR397b, and miR402	Response to water deficiency	[111]
White clover	DNA methylation	Stress memory phenomenon	[112]
Spinach and Arabidopsis	DNA methylation	Artificial induction of flowering	[113]
Maize	DNA methylation	Defense priming to herbivores and increase plant defense	[114]
Rapeseed and barley	Histone modifications	Favoring acceleration of crop breeding	[115]
Soybean	DNA methylation	Protective mechanism and seed development	[116]
Rice	DNA methylation	Artificial crossings	[117]
Rice	Downregulation of miR319c, miR164c, miR319b, and miR1861d	Response to drought	[118]
